# Crystal structures of 9-[bis­(benzyl­sulfan­yl)meth­yl]anthracene and of *cyclo*-dodeca­kis­(μ_2_-phenyl­methane­thiol­ato-κ^2^ *S*:*S*)hexa­palladium(6 *Pd*—*Pd*)–anthracene-9,10-dione (1/1)

**DOI:** 10.1107/S2056989021006113

**Published:** 2021-06-18

**Authors:** Abhinav Raghuvanshi, Anna Krupp, Lydie Viau, Michael Knorr, Carsten Strohmann

**Affiliations:** a Institut UTINAM UMR 6213 CNRS, Université Bourgogne Franche-Comté, 16, Route de Gray, 25030 Besançon, France; b Anorganische Chemie, TU Dortmund University, Otto-Hahn-Str. 6, D-44227 Dortmund, Germany

**Keywords:** crystal structure, cluster, palladium, thio­ether, thiol­ate, thio­acetal, supra­molecular network

## Abstract

The first title compound, C_29_H_24_S_2_, **L1**, represents an example of an anthracene-based functionalized di­thio­ether, which may be useful as a potential chelating or terminal ligand for coordination chemistry. This di­thio­acetal **L1** crystallizes in the monoclinic space group *P*2_1_/*c*. The phenyl rings of the benzyl groups and that of the anthracene unit form dihedral angles of 49.21 (4) and 58.79 (5)° and the crystal structure displays short C–H⋯π contacts. Surprisingly, when attempting to coordinate **L1** to [PdCl_2_(PhCN)_2_], instead of the targeted chelate complex [PdCl_2_(κ^2^-**L1**)], a cleavage reaction leads to the formation of the centrosymmetric hexa­nuclear cyclic cluster of composition [Pd_6_(μ_2_-SCH_2_Ph)_12_] **Pd6**, or [Pd_6_(C_7_H_7_S)_12_]·C_14_H_8_O_2_. This tiara-shaped hexa­mer crystallizing in the triclinic space group *P*  consists of six approximately square planar Pd(II)S_4_ centers, which are inter­connected through twelve μ_2_-bridging benzyl thiol­ate groups. The Pd⋯Pd contacts range from 3.0892 (2) to 3.1609 (2) Å and can be considered as weakly bonding. The unit cell of **Pd6** contains also a co-crystallized anthracene-9,10-dione mol­ecule.

## Abstract 2

The di­thio­actal bis­[(benzyl­thio)­meth­yl]anthracene has been synthesized and reacted with [PdCl_2_(PhCN)_2_] to yield the cyclic cluster [Pd_6_(μ_2_-SCH_2_Ph)_12_].

## Chemical context   

Acyclic and cyclic thio­acetals with the –S–C(*R*)(H)–S (*R* = H, alkyl, ar­yl) unit can either be synthesized by nucleophilic substitution of geminal dihalides *X*–C(*R*)(H)–*X* by thiol­ates *R*S^−^ (Murray *et al.*, 1981 ▸) or by reaction of aldehydes and ketones with thiols and di­thiols (Shaterian *et al.*, 2011 ▸). Because of their soft nature, organosulfur compounds preferentially inter­act with late transition metals in lower oxidation states. A variety of complexes as well as coordination polymers (CPs) of varying dimensionality, ranging from zero-dimensional (mol­ecular) to three-dimensional, have been synthesized using these types of di­thio­ether ligands and structurally characterized (Knaust & Keller, 2003 ▸; Awaleh *et al.*, 2005 ▸, 2008 ▸). However, many factors including the structural characteristics of the organic ligands, temperature, solvent, molar ratio, *etc*., greatly influence the formation of the resulting materials.

Over the last few years, we have been engaged in exploring the assembly of mol­ecular cluster compounds and coordination polymers using thio­ether ligands RSCH_2_S*R* (Peindy *et al.*, 2007 ▸; Knorr *et al.*, 2014 ▸; Schlachter *et al.*, 2020 ▸). Recently, we have also reported the synthesis of Cu^I^ coordination complexes ligated with cyclic thio­acetal ligands bearing various substituents (Raghuvanshi *et al.*, 2017 ▸, 2019 ▸; Schlachter *et al.*, 2018 ▸; Knauer *et al.*, 2020 ▸). Convenient synthetic protocols and inter­esting luminescent properties displayed by these complexes intrigued us to explore this field further.

Since the presence of an anthracene unit provides both rigidity as well as inter­esting luminescent properties to a given system, a large number of anthracene-based MOFs and CPs have been reported for various applications (for example: Hu *et al.*, 2020 ▸; Mohanty *et al.*, 2020 ▸; Quah *et al.*, 2016 ▸; Wang *et al.*, 2016 ▸). In most of these reports, either *N*- or *O*-donor substituents attached to the anthracene scaffold have been used as coordinating sites. In contrast, there are few reports where anthracene-based thio­ether ligands have been used for the construction of CPs. For example, a series of emissive mol­ecular compounds and CPs have been assembled by reaction of 9,10-bis­[(alkyl­thio)­meth­yl]anthracenes with Ag^I^ salts (Hu *et al.*, 2006 ▸). The synthesis of anthracene-based thio­acetals with different –S*R* substituents including **L1** has been briefly reported (Goswami *et al.*, 2008 ▸ and Shaterian *et al.*, 2011 ▸). However, no spectroscopic characterization data have been communicated. Furthermore, no examples of structurally characterized anthracene-based thio­acetals could be found within the Cambridge Structural Database. These disparities make this field inter­esting for further investigations.
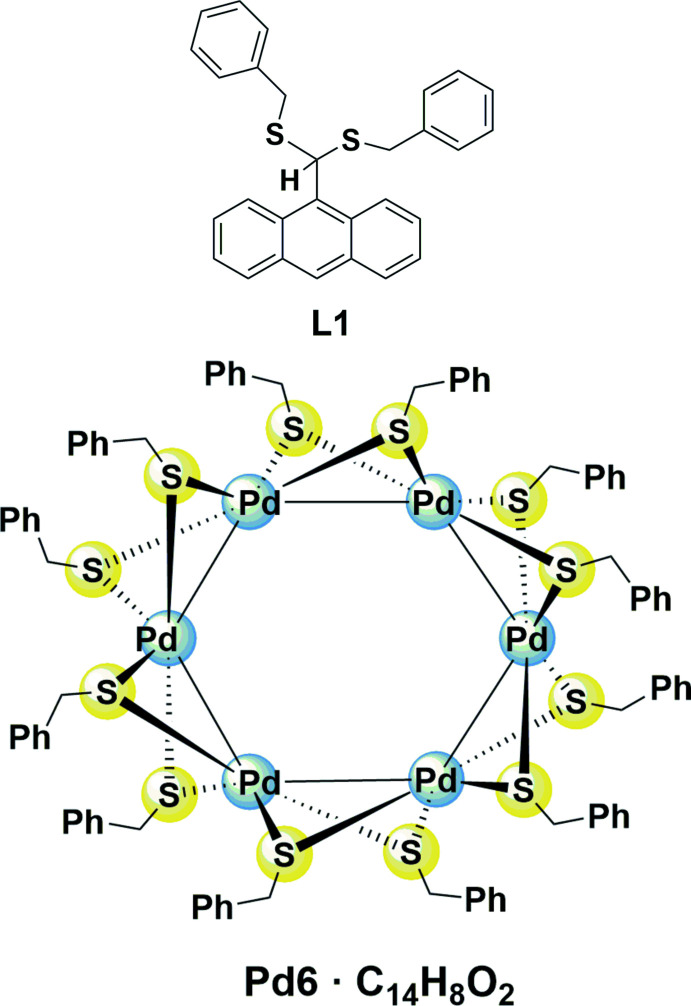


**Figure 1:**
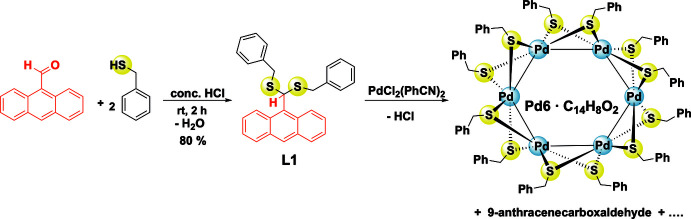
Synthesis scheme for **L1** and the cluster **Pd6·C_14_H_8_O_2_**

In this context, we synthesized the anthracene thio­acetal **L1** with the objective of using it as an *S*-donor ligand for the assembly of potentially luminescent coordination compounds. **L1** was prepared straightforwardly by the reaction of benzyl mercaptan and 9-anthracenecarboxaldehyde in the presence of an excess conc. HCl at room temperature (Fig. 1 ▸) and obtained in 80% yield as a yellow solid. Characteristic for its ^1^H NMR spectrum are two doublets at δ 3.55 and 3.79 ppm for the diastereotopic methyl­ene protons and a singlet at δ 5.94 ppm for the methine proton. The complete spectroscopic data are reported in the *Synthesis and crystallization* section.

With this starting material in hand, we attempted to ligate **L1** to [PdCl_2_(PhCN)_2_], (Fig. 1 ▸). Although the coordination chemistry of [PdCl_2_(S∩S)] compounds is dominated by chelate complexes in which open-chain di­thio­ether or macrocyclic polythio­ether ligands form five- or six-membered rings such as [PdCl_2_(1,2-bis­(phenyl­thio)­ethane-*S,S′*] (Rao *et al.*, 2015 ▸; Cambridge Structural Database refcode: CEYBUD01) or [PdCl_2_(1,4,7-tri­thia­cyclo­nonane-*S,S′*)] (GATLES; Blake *et al.*, 1988 ▸), there is just one structurally characterized example of a chelate complex [PdCl_2_(1,3,5,7-tetra­methyl-2,4,6,8,9,10-hexa­thia-adamantane-*S^4^,S^6^*)], in which the thia­macrocycle forms a strained four-membered chelate ring (DOCNOY; Pickardt & Rautenberg, 1986 ▸). It has also been reported that upon treatment of PhSCH_2_SPh with [M(MeCN)_4_][ClO_4_]_2_, the strained chelate complexes [*M*(PhSCH_2_SPh)_4_](ClO_4_)_2_ (*M* = Pd, Pt) are formed (Murray *et al.*, 1981 ▸). However, to our surprise, the targeted compound [PdCl_2_(anthracen-9-yl­methyl­ene)bis­(benzyl­sulfane)-*S,S′*)] was not formed according to the NMR data. Instead, a crystallographic study of a yellow–orange crystal revealed the formation of a cyclic hexa­nuclear thiol­ate-bridged cluster [Pd_6_(μ_2_-SCH_2_Ph)_12_], **Pd6**. It is well known that thio­acetals can be cleaved by soft Hg^II^ ions yielding aldehydes or other oxygenated products. One example is the HgI^II^-promoted deprotection of 3,5-bis­(di­thio­acetal)BODIPYs, in which cleavage of a di­thio­acetal function to aldehyde groups occurs (Madhu *et al.*, 2014 ▸). A mild qu­anti­tative AgNO_3_-promoted cleavage of fluorenenyl­ethanediyl-*S,S*-acetals with tri­chloro­isocyanuric acid yielding 9-fluorenone has also been reported (Olah *et al.*, 1980 ▸). We suppose that in our case PdCl_2_ behaves similarly, acting as electrophilic agent. We have not examined the mechanistic aspects of this unexpected reaction in detail, but the fact that **Pd6** co-crystallizes with one mol­ecule of anthracene-9,10-dione and smaller amounts of 9-anthraldehyde is in line with this hypothesis. It is noteworthy that this diketone has also been detected as one of the numerous oxidation products stemming from the oxidation of (anthracen-9-ylmeth­yl)(benz­yl)sulfane with ceric ammonium nitrate (Gopalakrishnan *et al.*, 2015 ▸).

Looking for a more rational manner to synthesize this tiara-like cluster, we attempted to prepare **Pd6** independently by reacting [PdCl_2_(PhCN)_2_] with 2.1 equivalents of benzyl mercaptan in CH_2_Cl_2_ solution. However, the isolation of **Pd6** was hampered by the co-crystallization of important amounts of the eight-membered cluster **Pd8** [Pd_8_(μ_2_-SCH_2_Ph)_16_], having a structure similar to that of [Pd_8_(μ_2_-SPr)_16_] (Higgins *et al.*, 1988 ▸). Details of this reaction will be reported elsewhere.

## Structural commentary   

**Figure 2:**
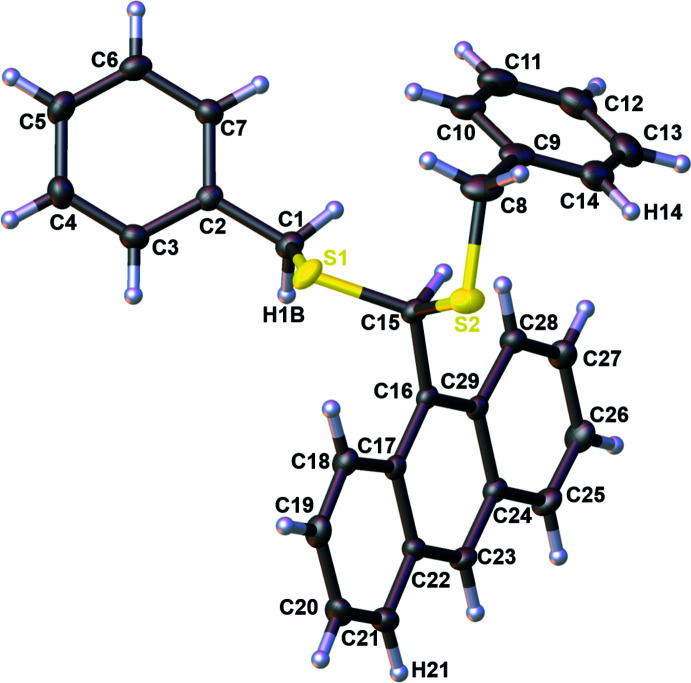
The mol­ecular structure of **L1** with atom labelling and displacement ellipsoids drawn at the 50% probability level.

Compound **L1** crystallizes from the mixed solvents CH_2_Cl_2_/hexane in the monoclinic crystal system with *P*2_1_/*c* space group. The mol­ecular structure of **L1** is presented in Fig. 2 ▸ and selected bond lengths and bond angles are given in Table 1 ▸. The C15—S1 and C15—S2 bond lengths of 1.8309 (12) and 1.8220 (12) Å are comparable with those of [BzSC(H)(C_6_H_4_NO_2_-*p*)SBz] (SUNMAQ) [1.8262 (19) and 1.818 (2) Å; Binkowska *et al.*, 2009 ▸], but are elongated compared with those of bis­(benzyl­sulfan­yl)methane (TUQPAX) [1.7988 (13) and 1.8013 (13) Å; Yang *et al.*, 2010 ▸) and 2-[bis­(benzyl­sulfan­yl)meth­yl]-6-meth­oxy­phenol (IGOBOY) [1.8132 (12) and 1.8189 (12) Å; Raghuvanshi *et al.*, 2020 ▸). The angle S1—C15—S2 of 110.93 (6)° in **L1** is wider than those of 4-nitro­phenyl­bis­(benzyl­sulfan­yl)methane [107.26 (6)°] and 2-[bis­(benzyl­sulfan­yl)meth­yl]-6-meth­oxy­phenol [107.76 (10)°], but considerably less than in [BzSCH_2_SBz] [117.33 (7)°]. The phenyl rings of the benzyl groups and that of the anthracene unit form dihedral angles of 49.21 (4) and 58.79 (5)°.

**Figure 3:**
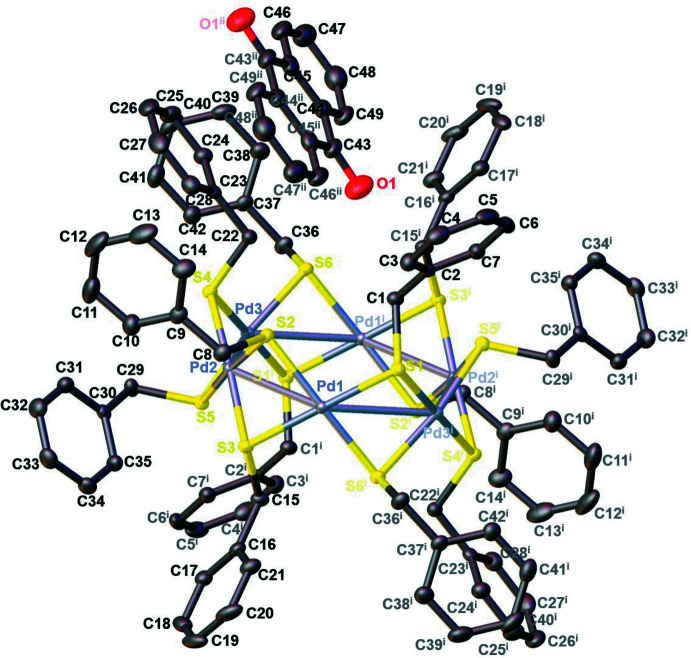
The mol­ecular structure of **Pd6·C_14_H_8_O_2_** with the atom labelling and displacement ellipsoids drawn at the 50% probability level [symmetry codes: (i) −*x* + 1, −*y* + 1, −*z* + 1; (ii) −*x* + 1, −*y* + 1, −*z* + 2]. The H atoms are not shown for clarity.

The inorganic part of the crystal structure of the reaction product of **L1** with [PdCl_2_(PhCN)_2_] shown in Fig. 3 ▸ is very similar overall to the structures of a series of other structurally characterized tiara-like hexa­nuclear clusters bridged by aliphatic thiol­ate groups such as [Pd_6_(μ_2_-SPr)_12_] (Kunchur, 1971 ▸; PDPRMC), [Pd_6_(μ_2_-SEt)_12_] (Stash *et al.*, 2001 ▸; UCIXAF), [Pd_6_(μ_2_-SCH_2_CH_2_OH)_12_] (Mahmudov *et al.*, 2013 ▸; XIPCUW), [Pd_6_(μ_2_-SBu)_12_] (Stash *et al.*, 2009 ▸; LAFBUR) and [Pd_6_(μ_2_-SHex­yl)_12_] (Ananikov *et al.*, 2012 ▸; FAVQEA). Furthermore, the structure of the thio­pheno­late-spanned compound [Pd_6_(μ_2_-SPh)_12_] has been reported (Stash *et al.*, 2009 ▸). However, within this series of metallacycles, the most reminiscent structure to our benzylic derivative [Pd_6_(μ_2_-SCH_2_Ph)_12_] is that of the phenyl­ethane­thiol­ate-decorated nanocluster [Pd_6_(μ_2_-SCH_2_CH_2_Ph)_12_] (Chen *et al.*, 2017 ▸; HEGPAN).

The core of **Pd6** consists of three crystallographically different Pd^II^ centers forming a centrosymmetric, almost planar, six-membered ring with Pd⋯Pd contacts ranging from 3.0892 (2) to 3.1609 (2) Å. The mean Pd⋯Pd separation of 3.1213 (2) Å is quite similar to that of the other derivatives and may be considered as weakly bonding (Stash *et al.*, 2009 ▸), being significantly shorter than the sum of the van der Waals radii for Pd (3.26 Å; Bondi, 1964 ▸). The mean separation of two symmetry-related opposite Pd nuclei is about 6.22 Å, the longest being that of 6.453 Å between Pd3 and Pd3′, justifying describing these compounds as nano-sized clusters. Each palladium atom is coordinated covalently to four μ_2_-sulfur atoms with an approximately square-planar geometry, and the average Pd—S bond length of 2.327 (5) Å is close to those of the other [Pd_6_(μ_2_-SR)_12_] analogues. The S—Pd—S bridge angles vary within the range 81.033 (16)–99.246 (16)°. The twelve sulfur atoms form two S_6_ hexa­gons parallel to the central Pd_6_ ring from both sides, conferring finally a tiara-like shape to the Pd_6_S_12_ scaffold.

Note that the crystal structure of anthracene-9,10-dione (also named 9,10-anthra­quinone) has already been the object of several crystallographic studies and is therefore not commented herein (Fu & Brock, 1998 ▸; Slouf, 2002 ▸).

## Supra­molecular features   

**Figure 4:**
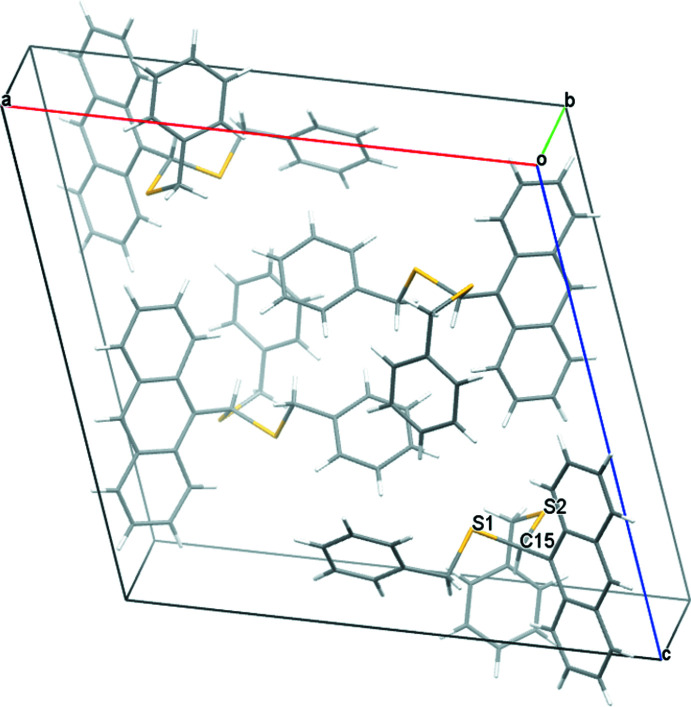
A view along the *b*-axis direction of the crystal packing of **L1**.

**Figure 5:**
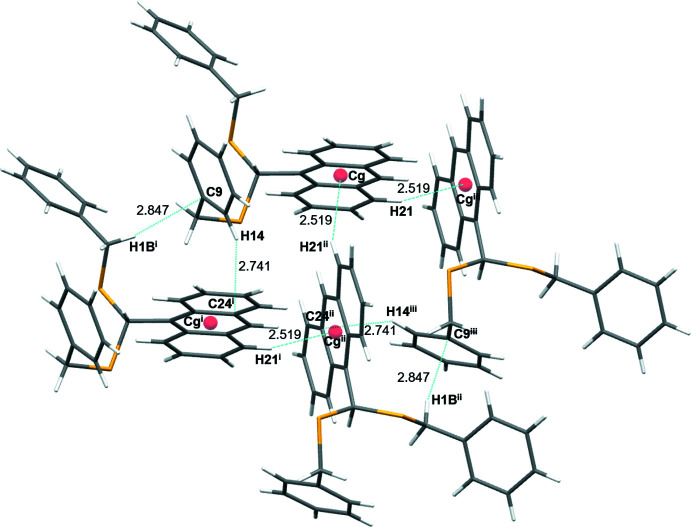
Inter­molecular C—H⋯π inter­actions occurring in **L1** generating a one-dimensional supra­molecular ribbon [symmetry codes: (i) *x*, *y* + 1, *z*; (ii) −*x* + 2, *y* + 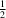, −*z* + 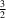; (iii) −*x* + 2, *y* − 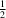, −*z* + 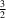]

The crystal packing of di­thio­actal **L1** is shown in Fig. 4 ▸. Three different types of C—H⋯π inter­actions are observed in the crystal structure **(**Fig. 5 ▸) where the H⋯π distances range from 2.51 to 2.84 Å. The C21—H21⋯*Cg*(C16/C17/C22/C23/C24/C29 centroid) distance of 2.519 (18) Å, the C14—H14⋯C24 distance of 2.741 (18) Å and the C1—H1*B*⋯C9 distance of 2.847 (16) Å are short enough to be considered as weak inter­molecular inter­actions (see Table 2 ▸). The closest C—H⋯S contact of 2.702 Å occurs between the aromatic H18 atom and S; however, the C18—H18⋯S1 angle of 123° suggests that this contribution has a neglectable impact on the conformation of **L1**.

**Figure 6:**
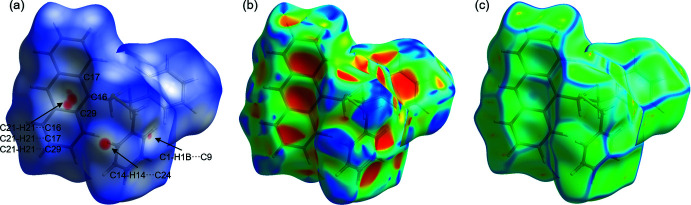
Hirshfeld surface mapped with (*a*) *d*_norm_, (*b*) shape-index and (*c*) curvedness for **L1**.

**Figure 8:**
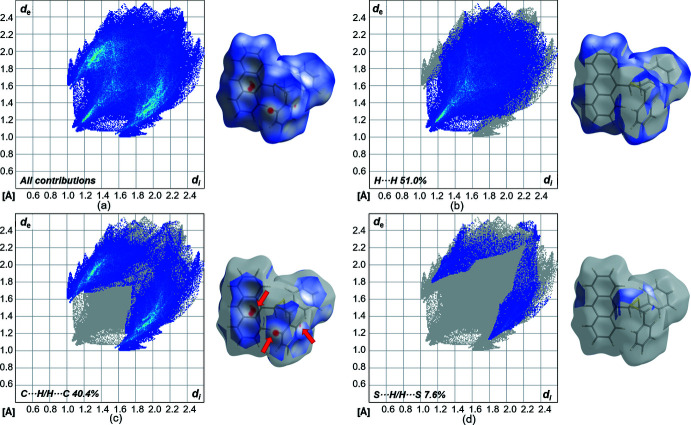
(*a*) Two-dimensional fingerprint plots of **L1**, showing all contributions, and delineated (*b*)–(*d*) showing the contributions of atoms within specific inter­acting pairs (blue areas).

A Hirshfeld surface analysis (Spackman & Jayatilaka, 2009 ▸) for the further investigation of close contacts and inter­molecular inter­actions was performed for **L1** using *CrystalExplorer17* (Turner *et al.*, 2017 ▸). Figs. 6 ▸ *a* and 7 ▸ illustrate the three-dimensional Hirshfeld surface mapped over *d*_norm_ in the range from −1.11 to 1.36 (arbitrary units). The red spots on the surface indicate the close contacts to adjacent mol­ecules. There are three areas of red spots which can be classified as C—H⋯π inter­actions. The first and most important inter­action is the C—H⋯π contact of one of the phenyl­methane­thiol­ate substituents to the anthracene scaffold of a neighboring mol­ecule (C14—H14⋯C24). Furthermore, there are significant inter­actions of the anthracene unit to an adjacent anthracene unit (C21—H21⋯C16/17/29). Then, there is also a weak C—H⋯π contact of two phenyl­methane­thiol­ate substituents (C1—H1*B*⋯C9). The contributions of the different types of inter­molecular inter­actions are shown in the two-dimensional fingerprint plots in Fig. 8 ▸. The weak van der Waals H⋯H contacts appear as the largest region with a 51.0% contribution. The C⋯H/H⋯C contacts exhibit a significant contribution at 40.4% and constitute a major contribution to the packing arrangement within the crystal structure. Fig. 6 ▸ *b* and 6*c* illustrate the Hirshfeld surface mapped over the shape-index and the curvedness. The shape-index shows large red regions of concave curvature for the anthracene motif, whereas the C—H-donors shows opposite curvature.

**Figure 9:**
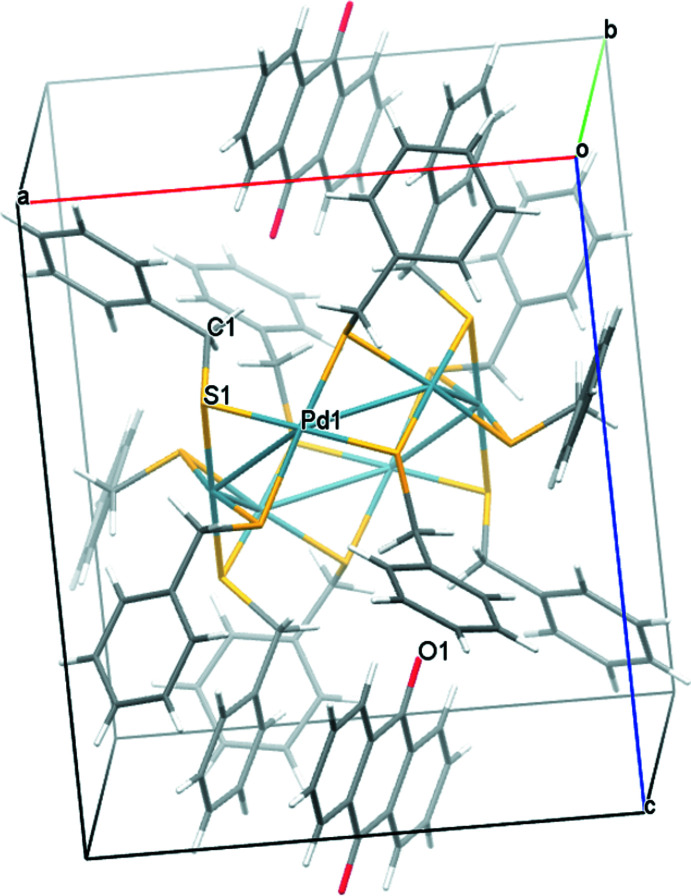
A view along the *b*-axis direction of the crystal packing of **Pd6·C_14_H_8_O_2_**.

Concerning the cluster **Pd6**, there are no particular directional inter­molecular inter­actions in the packing warranting any discussion. The packing is shown in Fig. 9 ▸.

## Database survey   

A search of the Cambridge Structural Database (Groom *et al.*, 2016 ▸) for related anthracene-substituted di­thio­acetals did not reveal any structure hits. However, there are several examples of mono­thio­ethers attached on an anthracenyl scaffold and include {9-[(2-chloro­eth­yl)thio]­meth­yl}anthracene (CETMAN; Lewis *et al.*, 1976 ▸), 1,6-bis­(9-anthr­yl)-2,5-di­thia­hexane (LEYHIH; Schwarze *et al.*, 2007 ▸) and *S*-(9-anthr­yl)methyl-3,5-di­nitro­thio­benzoate (VEZLUI; Fowelin *et al.*, 2007 ▸). A search for the bis­(benzyl­thio)­methane motif HC(SCH_2_Ph)_2_ revealed only three similar structures, namely 2,6,10,14,19,24-hexa-*p*-benz-4,8,12,16,17,21,22,26-octa­thia­tri­cyclo­(9.5.5.5^3,9^)hexa­cosa­phane benzene clathrate (CUHLUM; Takemura *et al.*, 1984 ▸), 4-nitro­phenyl-[bis­(benzyl­thio)]methane (SUNMAQ; Binkowska *et al.*, 2009 ▸) and 2-[bis(benzyl­sulfan­yl)meth­yl]-6-meth­oxy­phenol (IGOBOY; Raghuvanshi *et al.*, 2020 ▸).

In contrast to mononuclear palladium complexes bearing terminal phenyl­methane­thiol­ate groups such as *trans*-[Pd(SCH_2_Ph)_2_(PMe_3_)_2_] (Lee *et al.* 2015 ▸; NOQZOK), [Pd(SCH_2_Ph)_2_(1,2-bis­(di­phenyl­phosphino)ethane)] (Su *et al.* 1997*a* ▸,*b* ▸; TERREN) and [Pd(SCH_2_Ph)_2_(1,3-bis­(di­phenyl­phosphino)propane)] (Su *et al.* 1997 ▸ ▸; SUTMOJ), those of phenyl­methane­thiol­ate-bridged di- and polynuclear Pd complexes are scare. The only crystallographically characterized hit is the tetra­nuclear cluster [Pd_4_Se_4_(μ_2_-SCH_2_Ph)_2_(bis­(di­phenyl­phosphino)methane)Cl_2_] (Cao *et al.* 1998 ▸; JIXRAJ). The aforementioned [Pd_6_(μ_2_-S*R*)_12_] clusters have found applications as precursors for the preparation of monodisperse PdS nanoparticles (Yang *et al.*, 2007 ▸), for the self-assembly of palladiumthiol­ate bilayers (Thomas *et al.*, 2001 ▸), as fluorescence materials (Chen *et al.*, 2017 ▸) and as electrocatalysts for H and O evolution reactions (Gao & Chen, 2017 ▸). Also noteworthy is the observation that individual [Pd_6_(μ_2_-SCH_2_CH_2_OH)_12_] mol­ecules are inter­connected in the solid state by hydrogen bonds through the hy­droxy groups of the thiol­ate ligands, thus generating an infinite three-dimensional supra­molecular network (Mahmudov *et al.*, 2013 ▸). Concerning the influence of hydrogen-bonding interactions on nuclearity and structure for other tiara-like palladium complexes, see: Martin *et al.* (2018 ▸). Recently, a structurally related Pt^II^ thiol­ate complex [Pt_6_(μ_2_-SC_12_H_23_)_12_] has been prepared and probed as a macrocyclic host to include an Ag^I^ ion as guest (Shichibu *et al.*, 2016 ▸).

## Synthesis and crystallization   

9-Anthracenecarboxaldehyde (206 mg, 1 mmol) and benzyl mercaptan (348 mg, 3 mmol) were suspended in conc. HCl (2 ml) and allowed to stir at room temperature. After 2 h, the reaction mixture was neutralized with aqueous NaHCO_3_ solution and extracted with di­chloro­methane. The organic fraction was dried over Na_2_SO_4_, filtered and concentrated under reduced pressure. Purification by column chromatography using a hexa­ne/di­chloro­methane solvent mixture as eluent gives a pale-yellow solid product in 80% yield (350 mg). Crystals suitable for single-crystal X-ray crystallography were grown by slow diffusion of hexane into a di­chloro­methane solution of **L1**, m.p. 438–440 K. ^1^H NMR (400 MHz, δ in ppm, CD_2_Cl_2_): 9.03 (*dd*, *J* = 9.0 Hz, *J* = 1.1 Hz, 1H, H_18_), 8.39 (*s*, 1H, H_23_), 8.00 (*dd*, *J* = 8.5 Hz, *J* = 1.1 Hz, 1H, H_21_), 7.95 (*dd*, *J* = 8.5 Hz, *J* = 1.1 Hz, 1H, H_25_), 7.55–7.47 (*m*, 2H, H_19_, H_27_), *ddd* (*J* = 8.5 Hz, *J* = 6.5 Hz, *J* = 1.1 Hz, 1H, H_3_), 7.28–7.22 (*m*, 6H, H_Ph_ + H_6_), 7.14–7.09 (*m*, 5H, H_Ph_), 6.91 (*dd*, *J* = 9.0 Hz, *J* = 1.1 Hz, 1H, H_28_), 5.94 (*s*, 1H, CHS_2_), 3.79 (*d*, *J* = 13.7 Hz, 2H, CH_2_), 3.55 (*d*, *J* = 13.7 Hz, 2H, CH_2_). ^13^C{^1^H} NMR (101 MHz, δ in ppm, CD_2_Cl_2_) 138.34 (C_16_), 132.50 (C_17_), 131.46 (Cq), 131.36 (Cq), 130.28 (Cq), 129.58 (CHAr), 129.56 (C_21_), 129.47 (C_25_), 129.13 (Cq), 128.96 (CHAr), 128.84 (C_23_), 127.75 (C_18_), 127.53 (CHAr), 126.63 (C_26_), 125.61 (C_19_), 125.12 (C_20_), 124.91 (C_27_), 122.99 (C_28_), 45.02 (S_2_CH), 37.89 (SCH_2_). IR (ATR) cm ^−1^: 3050 and 3025 (C—H Ar), 2998, 2948 and 2906 (C—H aliphatic), 1589, 1519 (C=C), 696 (C—S).

**Reaction of L1 with PdCl_2_(PhCN)_2_**: **L1** (43 mg, 0.1 mmol) and PdCl_2_(PhCN)_2_ (38 mg, 0.1 mmol) were dissolved in 5 ml of di­chloro­methane and allowed to stir at room temperature for 30 minutes. During the reaction, a red solution was obtained, which was kept in refrigerator overnight yielding yellow crystals of 9-anthraldehyde along with yellow–orange co-crystals of the [Pd_6_(SCH_2_Ph)_12_·anthracene-9,10-dione] cluster, **Pd6**. ^1^H NMR (400 MHz, δ in ppm, CD_2_Cl_2_)): 8.92–6.86 (*m*, overlapping benzylic and anthracenyl H), 3.61 (*s*, SCH_2_), 3.58 (*s*, SCH_2_).

## Refinement   

Crystal data, data collection and structure refinement details are summarized in Table 3 ▸. For both compounds, the H atoms were positioned geometrically (C—H = 0.95–1.00 Å) and were refined using a riding model, with *U*_iso_(H) = 1.2*U*_eq_(C). Hydrogen atoms H1*B*, H14 and H21 for **L1** were located in the difference-Fourier map and refined freely.

## Supplementary Material

Crystal structure: contains datablock(s) mo_b0159_0m, mo_b0283_0m, New_Global_Publ_Block. DOI: 10.1107/S2056989021006113/hb7976sup1.cif

Structure factors: contains datablock(s) mo_b0159_0m. DOI: 10.1107/S2056989021006113/hb7976mo_b0159_0msup2.hkl

Structure factors: contains datablock(s) mo_b0283_0m. DOI: 10.1107/S2056989021006113/hb7976mo_b0283_0msup3.hkl

CCDC references: 2089413, 2089412

Additional supporting information:  crystallographic information; 3D view; checkCIF report

## Figures and Tables

**Figure 7:**
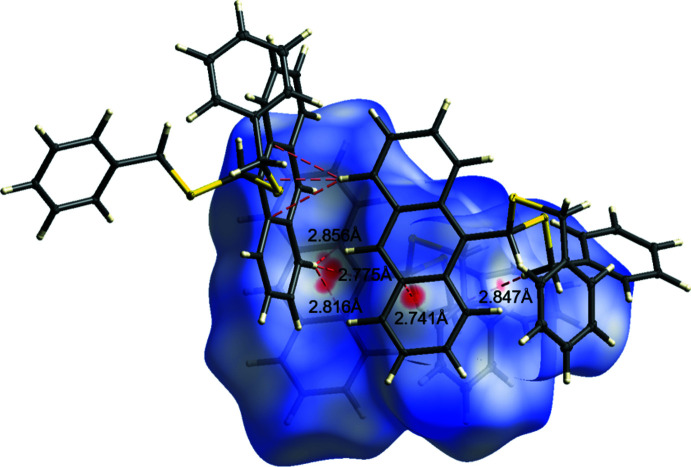
Hirshfeld surface analysis of **L1** showing close contacts in the crystal.

**Table 1:** Selected geometric parameters (Å, °) for **L1** [Chem scheme1]

S1—C15	1.8309 (12)	S2—C15	1.8220 (12)
			
S1—C15—S2	110.93 (6)		

**Table 2:** Close contacts (Å, °) for **L1** [Chem scheme1] *Cg* is the centroid of the C16/C17/C22–C24/C29 ring.

*D*—H⋯*A*	*D*—H	H⋯*A*	*D*⋯*A*	*D*—H⋯*A*
C21—H21⋯C16^i^	0.951 (17)	2.775 (17)	3.7095 (17)	167.6 (13)
C21—H21⋯C17^i^	0.951 (17)	2.856 (17)	3.7737 (18)	162.6 (13)
C21—H21⋯C29^i^	0.951 (17)	2.816 (17)	3.6338 (17)	144.7 (12)
C21—H21⋯*Cg* ^i^	0.951 (17)	2.519 (18)	3.4116 (14)	156.3 (13)
C14—H14⋯C24^ii^	0.976 (17)	2.741 (18)	3.5982 (19)	146.9 (13)
C1—H1*B*⋯C9^iii^	0.972 (16)	2.847 (16)	3.8023 (17)	168.0 (12)

Symmetry codes: (i) -x+2, y-{\script{1\over 2}}, -z+{\script{3\over 2}}; (ii) x, y+1, z; (iii) x, y-1, z.

**Table 3:** Experimental details

	**L1**	**Pd6**
Crystal data		
Chemical formula	C_29_H_24_S_2_	[Pd_6_(C_7_H_7_S)_12_]·C_14_H_8_O_2_
*M*_r_	436.60	2324.83
Crystal system, space group	Monoclinic, *P*2_1_/*c*	Triclinic, *P*\overline{1}
Temperature (K)	123	100
*a*, *b*, *c* (Å)	18.0842 (13), 7.5279 (5), 17.4975 (13)	12.4037 (6), 13.2255 (6), 14.7347 (7)
α, β, γ (°)	90, 108.439 (3), 90	109.842 (2), 91.616 (2), 91.191 (2)
*V* (Å^3^)	2259.7 (3)	2271.56 (19)
*Z*	4	1
Radiation type	Mo *K*α	Mo *K*α
μ (mm^−1^)	0.25	1.49
Crystal size (mm)	0.95 × 0.44 × 0.30	0.33 × 0.24 × 0.18

Data collection		
Diffractometer	Bruker D8 Venture	Bruker D8 Venture
Absorption correction	Multi-scan (*SADABS*; Bruker, 2016 ▸)	Multi-scan (*SADABS*; Bruker, 2016 ▸)
*T*_min_, *T*_max_	0.522, 0.563	0.300, 0.333
No. of measured, independent and observed [*I* > 2σ(*I*)] reflections	25688, 4994, 4423	109169, 10078, 9452
*R*_int_	0.025	0.028
(sin θ/λ)_max_ (Å^−1^)	0.641	0.644

Refinement		
*R*[*F*^2^ > 2σ(*F* ^2^)], *wR*(*F* ^2^), *S*	0.032, 0.086, 1.05	0.019, 0.048, 1.10
No. of reflections	4994	10078
No. of parameters	295	532
H-atom treatment	H atoms treated by a mixture of independent and constrained refinement	H-atom parameters constrained
Δρ_max_, Δρ_min_ (e Å^−3^)	0.24, −0.30	1.20, −0.79

Computer programs: *APEX2* (Bruker, 2018 ▸), *SAINT* (Bruker, 2016 ▸), *SHELXT* (Sheldrick, 2015*a* ▸), *SHELXL* (Sheldrick, 2015*b* ▸), *OLEX2* (Dolomanov *et al.*, 2009 ▸), *CrystalExplorer17* (Turner *et al.*, 2017 ▸), *publCIF* (Westrip, 2010 ▸) and *Mercury* (Macrae *et al.*, 2020 ▸).

## supplementary crystallographic information

### 9-[Bis(benzylsulfanyl)methyl]anthracene (mo_b0159_0m) Crystal data

**Table:**

C_29_H_24_S_2_	*F*(000) = 920
*M**_r_* = 436.60	*D*_x_ = 1.283 Mg m^−^^3^
Monoclinic, *P*2_1_/*c*	Mo *K*α radiation, λ = 0.71073 Å
*a* = 18.0842 (13) Å	Cell parameters from 9706 reflections
*b* = 7.5279 (5) Å	θ = 2.8–27.1°
*c* = 17.4975 (13) Å	µ = 0.25 mm^−^^1^
β = 108.439 (3)°	*T* = 123 K
*V* = 2259.7 (3) Å^3^	Block, yellow
*Z* = 4	0.95 × 0.44 × 0.30 mm

### 9-[Bis(benzylsulfanyl)methyl]anthracene (mo_b0159_0m) Data collection

**Table:**

Bruker D8 Venture diffractometer	4994 independent reflections
Radiation source: microfocus sealed X-ray tube, Incoatec Iµs	4423 reflections with *I* > 2σ(*I*)
HELIOS mirror optics monochromator	*R*_int_ = 0.025
Detector resolution: 10.4167 pixels mm^-1^	θ_max_ = 27.1°, θ_min_ = 2.8°
ω and φ scans	*h* = −23→23
Absorption correction: multi-scan (SADABS; Bruker, 2016)	*k* = −9→9
*T*_min_ = 0.522, *T*_max_ = 0.563	*l* = −22→22
25688 measured reflections	

### 9-[Bis(benzylsulfanyl)methyl]anthracene (mo_b0159_0m) Refinement

**Table:**

Refinement on *F*^2^	Primary atom site location: iterative
Least-squares matrix: full	Hydrogen site location: mixed
*R*[*F*^2^ > 2σ(*F*^2^)] = 0.032	H atoms treated by a mixture of independent and constrained refinement
*wR*(*F*^2^) = 0.086	*w* = 1/[σ^2^(*F*_o_^2^) + (0.0436*P*)^2^ + 0.9034*P*] where *P* = (*F*_o_^2^ + 2*F*_c_^2^)/3
*S* = 1.05	(Δ/σ)_max_ = 0.001
4994 reflections	Δρ_max_ = 0.24 e Å^−^^3^
295 parameters	Δρ_min_ = −0.30 e Å^−^^3^
0 restraints	

### 9-[Bis(benzylsulfanyl)methyl]anthracene (mo_b0159_0m) Special details

**Table:**

Geometry. All esds (except the esd in the dihedral angle between two l.s. planes) are estimated using the full covariance matrix. The cell esds are taken into account individually in the estimation of esds in distances, angles and torsion angles; correlations between esds in cell parameters are only used when they are defined by crystal symmetry. An approximate (isotropic) treatment of cell esds is used for estimating esds involving l.s. planes.

### 9-[Bis(benzylsulfanyl)methyl]anthracene (mo_b0159_0m) Fractional atomic coordinates and isotropic or equivalent isotropic displacement parameters (Å^2^)

**Table:**

	*x*	*y*	*z*	*U*_iso_*/*U*_eq_	
S1	0.68666 (2)	0.64448 (5)	0.70893 (2)	0.02319 (9)	
S2	0.80911 (2)	0.92465 (4)	0.72381 (2)	0.02454 (9)	
C1	0.62676 (7)	0.52418 (17)	0.62051 (8)	0.0205 (2)	
H1A	0.6175 (9)	0.602 (2)	0.5751 (9)	0.025*	
C2	0.55092 (7)	0.47989 (16)	0.63477 (7)	0.0185 (2)	
C3	0.54888 (7)	0.35800 (16)	0.69428 (8)	0.0221 (3)	
H3	0.5950	0.2966	0.7235	0.027*	
C4	0.47989 (8)	0.32580 (18)	0.71106 (8)	0.0269 (3)	
H4	0.4793	0.2443	0.7523	0.032*	
C5	0.41191 (8)	0.41281 (18)	0.66757 (8)	0.0270 (3)	
H5	0.3648	0.3909	0.6790	0.032*	
C6	0.41294 (7)	0.53190 (18)	0.60730 (8)	0.0250 (3)	
H6	0.3663	0.5899	0.5768	0.030*	
C7	0.48232 (7)	0.56614 (16)	0.59167 (7)	0.0213 (3)	
H7	0.4829	0.6494	0.5511	0.026*	
C8	0.73177 (9)	1.08485 (18)	0.67732 (9)	0.0324 (3)	
H8A	0.6879	1.0657	0.6987	0.039*	
H8B	0.7519	1.2066	0.6922	0.039*	
C9	0.70229 (8)	1.06923 (16)	0.58667 (9)	0.0269 (3)	
C10	0.62799 (8)	1.00169 (18)	0.54845 (9)	0.0298 (3)	
H10	0.5945	0.9749	0.5792	0.036*	
C11	0.60257 (9)	0.97328 (19)	0.46564 (9)	0.0350 (3)	
H11	0.5521	0.9259	0.4402	0.042*	
C12	0.65053 (10)	1.0137 (2)	0.42027 (9)	0.0368 (3)	
H12	0.6332	0.9937	0.3638	0.044*	
C13	0.72407 (10)	1.08361 (19)	0.45753 (10)	0.0378 (4)	
H13	0.7568	1.1134	0.4264	0.045*	
C14	0.74995 (9)	1.11021 (18)	0.54031 (10)	0.0330 (3)	
C15	0.76483 (7)	0.72278 (16)	0.67172 (7)	0.0177 (2)	
H15	0.7393	0.7570	0.6142	0.021*	
C16	0.82754 (6)	0.59003 (15)	0.67177 (7)	0.0151 (2)	
C17	0.87649 (7)	0.51631 (15)	0.74463 (7)	0.0160 (2)	
C18	0.86799 (7)	0.55321 (17)	0.82190 (7)	0.0203 (2)	
H18	0.8266	0.6274	0.8251	0.024*	
C19	0.91823 (8)	0.48387 (18)	0.89085 (7)	0.0240 (3)	
H19	0.9110	0.5098	0.9411	0.029*	
C20	0.98118 (8)	0.37349 (18)	0.88869 (8)	0.0251 (3)	
H20	1.0163	0.3281	0.9373	0.030*	
C21	0.99092 (8)	0.33320 (17)	0.81703 (8)	0.0231 (3)	
C22	0.93948 (7)	0.40166 (15)	0.74321 (7)	0.0178 (2)	
C23	0.95162 (7)	0.36306 (16)	0.67019 (7)	0.0196 (2)	
H23	0.9933	0.2866	0.6697	0.024*	
C24	0.90401 (7)	0.43409 (15)	0.59806 (7)	0.0172 (2)	
C25	0.91875 (7)	0.39569 (17)	0.52438 (8)	0.0228 (3)	
H25	0.9608	0.3194	0.5251	0.027*	
C26	0.87412 (8)	0.46550 (19)	0.45322 (8)	0.0257 (3)	
H26	0.8849	0.4387	0.4048	0.031*	
C27	0.81107 (8)	0.57903 (18)	0.45207 (7)	0.0239 (3)	
H27	0.7797	0.6278	0.4023	0.029*	
C28	0.79469 (7)	0.61934 (16)	0.52090 (7)	0.0203 (2)	
H28	0.7520	0.6954	0.5180	0.024*	
C29	0.84043 (6)	0.54966 (15)	0.59774 (7)	0.0154 (2)	
H1B	0.6540 (9)	0.417 (2)	0.6137 (9)	0.025 (4)*	
H21	1.0321 (9)	0.258 (2)	0.8136 (9)	0.033 (4)*	
H14	0.8029 (10)	1.152 (2)	0.5669 (10)	0.038 (5)*	

### 9-[Bis(benzylsulfanyl)methyl]anthracene (mo_b0159_0m) Atomic displacement parameters (Å^2^)

**Table:**

	*U*^11^	*U*^22^	*U*^33^	*U*^12^	*U*^13^	*U*^23^
S1	0.01573 (15)	0.03525 (18)	0.02062 (16)	−0.00149 (12)	0.00864 (12)	−0.00719 (12)
S2	0.02187 (17)	0.02029 (16)	0.02713 (17)	0.00168 (11)	0.00160 (13)	−0.00742 (12)
C1	0.0191 (6)	0.0225 (6)	0.0207 (6)	0.0010 (5)	0.0076 (5)	−0.0028 (5)
C2	0.0183 (6)	0.0188 (5)	0.0187 (6)	−0.0005 (4)	0.0060 (5)	−0.0038 (4)
C3	0.0209 (6)	0.0208 (6)	0.0228 (6)	0.0017 (5)	0.0043 (5)	0.0017 (5)
C4	0.0283 (7)	0.0261 (6)	0.0266 (6)	−0.0042 (5)	0.0093 (5)	0.0040 (5)
C5	0.0208 (6)	0.0304 (7)	0.0311 (7)	−0.0060 (5)	0.0102 (5)	−0.0030 (6)
C6	0.0175 (6)	0.0260 (6)	0.0286 (7)	0.0018 (5)	0.0032 (5)	−0.0016 (5)
C7	0.0221 (6)	0.0202 (6)	0.0202 (6)	0.0004 (5)	0.0047 (5)	0.0009 (5)
C8	0.0315 (7)	0.0227 (6)	0.0377 (8)	0.0088 (6)	0.0032 (6)	−0.0093 (6)
C9	0.0253 (7)	0.0145 (6)	0.0364 (7)	0.0061 (5)	0.0032 (6)	−0.0024 (5)
C10	0.0222 (6)	0.0246 (6)	0.0399 (8)	0.0062 (5)	0.0058 (6)	0.0022 (6)
C11	0.0276 (7)	0.0288 (7)	0.0388 (8)	0.0039 (6)	−0.0033 (6)	0.0016 (6)
C12	0.0449 (9)	0.0272 (7)	0.0324 (8)	0.0068 (6)	0.0039 (7)	0.0053 (6)
C13	0.0439 (9)	0.0263 (7)	0.0453 (9)	0.0049 (6)	0.0171 (7)	0.0116 (6)
C14	0.0282 (7)	0.0192 (6)	0.0479 (9)	−0.0019 (5)	0.0068 (7)	0.0025 (6)
C15	0.0156 (5)	0.0190 (5)	0.0188 (5)	0.0003 (4)	0.0058 (4)	−0.0032 (4)
C16	0.0133 (5)	0.0154 (5)	0.0177 (5)	−0.0017 (4)	0.0064 (4)	−0.0015 (4)
C17	0.0147 (5)	0.0168 (5)	0.0177 (6)	−0.0018 (4)	0.0066 (4)	−0.0005 (4)
C18	0.0195 (6)	0.0244 (6)	0.0187 (6)	−0.0010 (5)	0.0083 (5)	−0.0018 (5)
C19	0.0256 (6)	0.0312 (7)	0.0172 (6)	−0.0034 (5)	0.0095 (5)	0.0001 (5)
C20	0.0227 (6)	0.0310 (7)	0.0195 (6)	−0.0001 (5)	0.0036 (5)	0.0082 (5)
C21	0.0203 (6)	0.0243 (6)	0.0243 (6)	0.0042 (5)	0.0064 (5)	0.0054 (5)
C22	0.0162 (6)	0.0174 (5)	0.0195 (6)	0.0004 (4)	0.0052 (5)	0.0022 (4)
C23	0.0163 (6)	0.0200 (6)	0.0235 (6)	0.0036 (4)	0.0077 (5)	−0.0008 (5)
C24	0.0158 (5)	0.0178 (5)	0.0189 (6)	−0.0011 (4)	0.0068 (4)	−0.0024 (4)
C25	0.0201 (6)	0.0287 (6)	0.0220 (6)	0.0026 (5)	0.0101 (5)	−0.0045 (5)
C26	0.0259 (7)	0.0357 (7)	0.0178 (6)	−0.0006 (6)	0.0102 (5)	−0.0041 (5)
C27	0.0224 (6)	0.0312 (7)	0.0171 (6)	0.0000 (5)	0.0047 (5)	0.0021 (5)
C28	0.0176 (6)	0.0228 (6)	0.0202 (6)	0.0014 (5)	0.0057 (5)	0.0010 (5)
C29	0.0137 (5)	0.0155 (5)	0.0175 (5)	−0.0024 (4)	0.0056 (4)	−0.0015 (4)

### 9-[Bis(benzylsulfanyl)methyl]anthracene (mo_b0159_0m) Geometric parameters (Å, º)

**Table:**

S1—C1	1.8240 (13)	C13—C14	1.389 (2)
S1—C15	1.8309 (12)	C14—H14	0.976 (17)
S2—C8	1.8309 (14)	C15—H15	1.0000
S2—C15	1.8220 (12)	C15—C16	1.5114 (15)
C1—H1A	0.958 (16)	C16—C17	1.4153 (16)
C1—C2	1.5070 (17)	C16—C29	1.4197 (16)
C1—H1B	0.971 (16)	C17—C18	1.4355 (16)
C2—C3	1.3971 (18)	C17—C22	1.4356 (16)
C2—C7	1.3920 (17)	C18—H18	0.9500
C3—H3	0.9500	C18—C19	1.3639 (18)
C3—C4	1.3905 (19)	C19—H19	0.9500
C4—H4	0.9500	C19—C20	1.4195 (19)
C4—C5	1.3884 (19)	C20—H20	0.9500
C5—H5	0.9500	C20—C21	1.3545 (19)
C5—C6	1.3887 (19)	C21—C22	1.4281 (17)
C6—H6	0.9500	C21—H21	0.951 (17)
C6—C7	1.3902 (18)	C22—C23	1.3937 (17)
C7—H7	0.9500	C23—H23	0.9500
C8—H8A	0.9900	C23—C24	1.3902 (17)
C8—H8B	0.9900	C24—C25	1.4262 (16)
C8—C9	1.510 (2)	C24—C29	1.4405 (16)
C9—C10	1.3938 (19)	C25—H25	0.9500
C9—C14	1.392 (2)	C25—C26	1.3573 (19)
C10—H10	0.9500	C26—H26	0.9500
C10—C11	1.391 (2)	C26—C27	1.4200 (19)
C11—H11	0.9500	C27—H27	0.9500
C11—C12	1.382 (2)	C27—C28	1.3623 (18)
C12—H12	0.9500	C28—H28	0.9500
C12—C13	1.386 (2)	C28—C29	1.4365 (16)
C13—H13	0.9500		
			
C1—S1—C15	100.16 (6)	C13—C14—H14	120.0 (10)
C15—S2—C8	100.02 (6)	S1—C15—H15	106.2
S1—C1—H1A	107.5 (9)	S1—C15—S2	110.93 (6)
S1—C1—H1B	109.1 (9)	S2—C15—H15	106.2
H1A—C1—H1B	111.7 (13)	C16—C15—S1	116.78 (8)
C2—C1—S1	107.27 (8)	C16—C15—S2	109.89 (8)
C2—C1—H1A	110.1 (9)	C16—C15—H15	106.2
C2—C1—H1B	111.0 (9)	C17—C16—C15	121.01 (10)
C3—C2—C1	120.58 (11)	C17—C16—C29	120.10 (10)
C7—C2—C1	120.60 (11)	C29—C16—C15	118.73 (10)
C7—C2—C3	118.75 (11)	C16—C17—C18	123.35 (11)
C2—C3—H3	119.7	C16—C17—C22	119.59 (10)
C4—C3—C2	120.59 (12)	C18—C17—C22	117.04 (11)
C4—C3—H3	119.7	C17—C18—H18	119.4
C3—C4—H4	120.0	C19—C18—C17	121.28 (12)
C5—C4—C3	120.01 (12)	C19—C18—H18	119.4
C5—C4—H4	120.0	C18—C19—H19	119.5
C4—C5—H5	120.0	C18—C19—C20	121.06 (12)
C4—C5—C6	119.91 (12)	C20—C19—H19	119.5
C6—C5—H5	120.0	C19—C20—H20	120.1
C5—C6—H6	120.0	C21—C20—C19	119.74 (12)
C5—C6—C7	119.92 (12)	C21—C20—H20	120.1
C7—C6—H6	120.0	C20—C21—C22	121.21 (12)
C2—C7—H7	119.6	C20—C21—H21	121.7 (10)
C6—C7—C2	120.81 (12)	C22—C21—H21	117.1 (10)
C6—C7—H7	119.6	C21—C22—C17	119.65 (11)
S2—C8—H8A	109.1	C23—C22—C17	119.81 (11)
S2—C8—H8B	109.1	C23—C22—C21	120.50 (11)
H8A—C8—H8B	107.8	C22—C23—H23	119.3
C9—C8—S2	112.44 (9)	C24—C23—C22	121.35 (11)
C9—C8—H8A	109.1	C24—C23—H23	119.3
C9—C8—H8B	109.1	C23—C24—C25	120.23 (11)
C10—C9—C8	119.94 (14)	C23—C24—C29	120.02 (11)
C14—C9—C8	121.12 (13)	C25—C24—C29	119.74 (11)
C14—C9—C10	118.80 (14)	C24—C25—H25	119.2
C9—C10—H10	119.8	C26—C25—C24	121.59 (12)
C11—C10—C9	120.45 (14)	C26—C25—H25	119.2
C11—C10—H10	119.8	C25—C26—H26	120.4
C10—C11—H11	119.9	C25—C26—C27	119.19 (11)
C12—C11—C10	120.24 (14)	C27—C26—H26	120.4
C12—C11—H11	119.9	C26—C27—H27	119.3
C11—C12—H12	120.1	C28—C27—C26	121.32 (12)
C11—C12—C13	119.75 (15)	C28—C27—H27	119.3
C13—C12—H12	120.1	C27—C28—H28	119.2
C12—C13—H13	119.9	C27—C28—C29	121.61 (11)
C12—C13—C14	120.13 (15)	C29—C28—H28	119.2
C14—C13—H13	119.9	C16—C29—C24	119.14 (10)
C9—C14—H14	119.3 (10)	C16—C29—C28	124.31 (11)
C13—C14—C9	120.61 (14)	C28—C29—C24	116.54 (10)
			
S1—C1—C2—C3	−67.96 (13)	C15—C16—C29—C24	175.17 (10)
S1—C1—C2—C7	108.83 (11)	C15—C16—C29—C28	−3.66 (17)
S1—C15—C16—C17	−63.16 (13)	C16—C17—C18—C19	−177.73 (12)
S1—C15—C16—C29	121.33 (10)	C16—C17—C22—C21	177.36 (11)
S2—C8—C9—C10	109.50 (13)	C16—C17—C22—C23	−0.39 (17)
S2—C8—C9—C14	−66.21 (15)	C17—C16—C29—C24	−0.38 (16)
S2—C15—C16—C17	64.29 (12)	C17—C16—C29—C28	−179.21 (11)
S2—C15—C16—C29	−111.22 (10)	C17—C18—C19—C20	0.44 (19)
C1—S1—C15—S2	153.75 (6)	C17—C22—C23—C24	0.29 (18)
C1—S1—C15—C16	−79.31 (9)	C18—C17—C22—C21	−1.20 (17)
C1—C2—C3—C4	175.75 (11)	C18—C17—C22—C23	−178.95 (11)
C1—C2—C7—C6	−176.90 (11)	C18—C19—C20—C21	−1.3 (2)
C2—C3—C4—C5	1.1 (2)	C19—C20—C21—C22	0.8 (2)
C3—C2—C7—C6	−0.05 (18)	C20—C21—C22—C17	0.42 (19)
C3—C4—C5—C6	0.0 (2)	C20—C21—C22—C23	178.16 (12)
C4—C5—C6—C7	−1.2 (2)	C21—C22—C23—C24	−177.45 (11)
C5—C6—C7—C2	1.19 (19)	C22—C17—C18—C19	0.77 (17)
C7—C2—C3—C4	−1.10 (19)	C22—C23—C24—C25	178.59 (11)
C8—S2—C15—S1	−73.63 (8)	C22—C23—C24—C29	−0.23 (18)
C8—S2—C15—C16	155.73 (9)	C23—C24—C25—C26	−178.85 (12)
C8—C9—C10—C11	−174.81 (12)	C23—C24—C29—C16	0.27 (17)
C8—C9—C14—C13	175.49 (13)	C23—C24—C29—C28	179.19 (11)
C9—C10—C11—C12	−0.7 (2)	C24—C25—C26—C27	−0.2 (2)
C10—C9—C14—C13	−0.3 (2)	C25—C24—C29—C16	−178.55 (11)
C10—C11—C12—C13	−0.3 (2)	C25—C24—C29—C28	0.37 (16)
C11—C12—C13—C14	1.0 (2)	C25—C26—C27—C28	0.1 (2)
C12—C13—C14—C9	−0.8 (2)	C26—C27—C28—C29	0.2 (2)
C14—C9—C10—C11	1.00 (19)	C27—C28—C29—C16	178.40 (12)
C15—S1—C1—C2	−169.19 (8)	C27—C28—C29—C24	−0.46 (17)
C15—S2—C8—C9	−47.89 (12)	C29—C16—C17—C18	178.91 (11)
C15—C16—C17—C18	3.46 (17)	C29—C16—C17—C22	0.44 (16)
C15—C16—C17—C22	−175.01 (10)	C29—C24—C25—C26	−0.04 (19)

### 9-[Bis(benzylsulfanyl)methyl]anthracene (mo_b0159_0m) Hydrogen-bond geometry (Å, º)

*Cg* is the centroid of the C16/C17/C22–C24/C29 ring.

**Table:**

*D*—H···*A*	*D*—H	H···*A*	*D*···*A*	*D*—H···*A*
C21—H21···C16^i^	0.951 (17)	2.775 (17)	3.7095 (17)	167.6 (13)
C21—H21···C17^i^	0.951 (17)	2.856 (17)	3.7737 (18)	162.6 (13)
C21—H21···C29^i^	0.951 (17)	2.816 (17)	3.6338 (17)	144.7 (12)
C21—H21···*Cg*^i^	0.951 (17)	2.519 (18)	3.4116 (14)	156.3 (13)
C14—H14···C24^ii^	0.976 (17)	2.741 (18)	3.5982 (19)	146.9 (13)
C1—H1*B*···C9^iii^	0.972 (16)	2.847 (16)	3.8023 (17)	168.0 (12)

Symmetry codes: (i) −*x*+2, *y*−1/2, −*z*+3/2; (ii) *x*, *y*+1, *z*; (iii) *x*, *y*−1, *z*.

### cyclo-Dodecakis(µ2-phenylmethanethiolato-κ2S:S)hexapalladium(6 Pd—Pd)–anthracene-9,10-dione (1/1) (mo_b0283_0m) Crystal data

**Table:**

[Pd_6_(C_7_H_7_S)_12_]·C_14_H_8_O_2_	*Z* = 1
*M**_r_* = 2324.83	*F*(000) = 1164
Triclinic, *P*1	*D*_x_ = 1.699 Mg m^−^^3^
*a* = 12.4037 (6) Å	Mo *K*α radiation, λ = 0.71073 Å
*b* = 13.2255 (6) Å	Cell parameters from 9790 reflections
*c* = 14.7347 (7) Å	θ = 2.4–27.2°
α = 109.842 (2)°	µ = 1.49 mm^−^^1^
β = 91.616 (2)°	*T* = 100 K
γ = 91.191 (2)°	Block, yellow
*V* = 2271.56 (19) Å^3^	0.33 × 0.24 × 0.18 mm

### cyclo-Dodecakis(µ2-phenylmethanethiolato-κ2S:S)hexapalladium(6 Pd—Pd)–anthracene-9,10-dione (1/1) (mo_b0283_0m) Data collection

**Table:**

Bruker D8 Venture diffractometer	10078 independent reflections
Radiation source: microfocus sealed X-ray tube, Incoatec Iµs	9452 reflections with *I* > 2σ(*I*)
HELIOS mirror optics monochromator	*R*_int_ = 0.028
Detector resolution: 10.4167 pixels mm^-1^	θ_max_ = 27.3°, θ_min_ = 2.3°
φ and ω scans	*h* = −15→15
Absorption correction: multi-scan (SADABS; Bruker, 2016)	*k* = −16→16
*T*_min_ = 0.300, *T*_max_ = 0.333	*l* = −18→18
109169 measured reflections	

### cyclo-Dodecakis(µ2-phenylmethanethiolato-κ2S:S)hexapalladium(6 Pd—Pd)–anthracene-9,10-dione (1/1) (mo_b0283_0m) Refinement

**Table:**

Refinement on *F*^2^	Primary atom site location: dual
Least-squares matrix: full	Hydrogen site location: inferred from neighbouring sites
*R*[*F*^2^ > 2σ(*F*^2^)] = 0.019	H-atom parameters constrained
*wR*(*F*^2^) = 0.048	*w* = 1/[σ^2^(*F*_o_^2^) + (0.0198*P*)^2^ + 2.3315*P*] where *P* = (*F*_o_^2^ + 2*F*_c_^2^)/3
*S* = 1.10	(Δ/σ)_max_ = 0.003
10078 reflections	Δρ_max_ = 1.20 e Å^−^^3^
532 parameters	Δρ_min_ = −0.79 e Å^−^^3^
0 restraints	

### cyclo-Dodecakis(µ2-phenylmethanethiolato-κ2S:S)hexapalladium(6 Pd—Pd)–anthracene-9,10-dione (1/1) (mo_b0283_0m) Special details

**Table:**

Geometry. All esds (except the esd in the dihedral angle between two l.s. planes) are estimated using the full covariance matrix. The cell esds are taken into account individually in the estimation of esds in distances, angles and torsion angles; correlations between esds in cell parameters are only used when they are defined by crystal symmetry. An approximate (isotropic) treatment of cell esds is used for estimating esds involving l.s. planes.

### cyclo-Dodecakis(µ2-phenylmethanethiolato-κ2S:S)hexapalladium(6 Pd—Pd)–anthracene-9,10-dione (1/1) (mo_b0283_0m) Fractional atomic coordinates and isotropic or equivalent isotropic displacement parameters (Å^2^)

**Table:**

	*x*	*y*	*z*	*U*_iso_*/*U*_eq_	
Pd1	0.44100 (2)		0.57757 (2)	0.01264 (3)	
Pd2	0.67651 (2)		0.61284 (2)	0.01273 (4)	
Pd3	0.74009 (2)		0.53617 (2)	0.01294 (4)	
S1	0.27073 (4)		0.62062 (3)	0.01475 (8)	
S2	0.53960 (4)		0.71742 (3)	0.01514 (8)	
S3	0.61070 (4)		0.53538 (3)	0.01522 (9)	
S4	0.74917 (4)		0.69054 (3)	0.01469 (8)	
S5	0.79906 (4)		0.49857 (3)	0.01519 (9)	
S6	0.65044 (4)		0.56887 (3)	0.01522 (9)	
C1	0.28536 (15)		0.71060 (13)	0.0183 (4)	
H1A	0.2914		0.6773	0.022*	
H1B	0.3521		0.7508	0.022*	
C2	0.18968 (15)		0.77377 (13)	0.0183 (4)	
C3	0.18149 (16)		0.84843 (14)	0.0232 (4)	
H3	0.2364		0.8586	0.028*	
C4	0.09401 (19)		0.90784 (15)	0.0306 (5)	
H4	0.0897		0.9589	0.037*	
C5	0.01266 (18)		0.89271 (17)	0.0344 (5)	
H5	−0.0477		0.9330	0.041*	
C6	0.01964 (17)		0.81929 (17)	0.0307 (5)	
H6	−0.0359		0.8090	0.037*	
C7	0.10780 (16)		0.75997 (15)	0.0229 (4)	
H7	0.1121		0.7096	0.027*	
C8	0.57745 (16)		0.77333 (14)	0.0200 (4)	
H8A	0.6094		0.7244	0.024*	
H8B	0.5121		0.7966	0.024*	
C9	0.65716 (16)		0.85634 (14)	0.0204 (4)	
C10	0.76764 (18)		0.84224 (17)	0.0292 (5)	
H10	0.7929		0.7797	0.035*	
C11	0.8405 (2)		0.9192 (2)	0.0423 (6)	
H11	0.9157		0.9094	0.051*	
C12	0.8046 (2)		1.0104 (2)	0.0457 (7)	
H12	0.8550		1.0630	0.055*	
C13	0.6952 (2)		1.02487 (17)	0.0410 (6)	
H13	0.6705		1.0875	0.049*	
C14	0.6216 (2)		0.94840 (15)	0.0286 (5)	
H14	0.5465		0.9586	0.034*	
C15	0.62522 (16)		0.40406 (13)	0.0186 (4)	
H15A	0.6840		0.3891	0.022*	
H15B	0.5575		0.3726	0.022*	
C16	0.65046 (15)		0.36623 (13)	0.0173 (4)	
C17	0.74129 (16)		0.31192 (14)	0.0217 (4)	
H17	0.7869		0.2981	0.026*	
C18	0.76570 (19)		0.27772 (15)	0.0297 (5)	
H18	0.8279		0.2408	0.036*	
C19	0.6997 (2)		0.29739 (15)	0.0346 (6)	
H19	0.7169		0.2751	0.042*	
C20	0.6084 (2)		0.34971 (15)	0.0317 (5)	
H20	0.5623		0.3621	0.038*	
C21	0.58351 (18)		0.38428 (14)	0.0236 (4)	
H21	0.5207		0.4204	0.028*	
C22	0.65300 (15)		0.77154 (13)	0.0173 (4)	
H22A	0.5908		0.7803	0.021*	
H22B	0.6257		0.7433	0.021*	
C23	0.71036 (15)		0.86724 (13)	0.0170 (4)	
C24	0.76401 (17)		0.88386 (15)	0.0238 (4)	
H24	0.7644		0.8345	0.029*	
C25	0.81688 (19)		0.97204 (17)	0.0337 (5)	
H25	0.8541		0.9827	0.040*	
C26	0.8154 (2)		1.04440 (16)	0.0379 (6)	
H26	0.8503		1.1053	0.046*	
C27	0.7634 (2)		1.02844 (16)	0.0351 (5)	
H27	0.7632		1.0781	0.042*	
C28	0.71125 (17)		0.93983 (15)	0.0250 (4)	
H28	0.6760		0.9289	0.030*	
C29	0.93066 (15)		0.55928 (15)	0.0193 (4)	
H29A	0.9376		0.6184	0.023*	
H29B	0.9887		0.5161	0.023*	
C30	0.94446 (14)		0.58605 (13)	0.0161 (3)	
C31	0.99352 (16)		0.67410 (14)	0.0219 (4)	
H31	1.0152		0.7183	0.026*	
C32	1.01116 (18)		0.69817 (15)	0.0271 (4)	
H32	1.0455		0.7585	0.033*	
C33	0.97909 (17)		0.63512 (16)	0.0249 (4)	
H33	0.9920		0.6516	0.030*	
C34	0.92801 (17)		0.54779 (15)	0.0237 (4)	
H34	0.9045		0.5046	0.028*	
C35	0.91107 (16)		0.52327 (14)	0.0206 (4)	
H35	0.8763		0.4630	0.025*	
C36	0.74754 (16)		0.55010 (14)	0.0204 (4)	
H36A	0.7085		0.5319	0.025*	
H36B	0.7954		0.4971	0.025*	
C37	0.81360 (15)		0.64207 (14)	0.0185 (4)	
C38	0.78166 (18)		0.70989 (15)	0.0257 (4)	
H38	0.7179		0.6978	0.031*	
C39	0.8426 (2)		0.79530 (16)	0.0317 (5)	
H39	0.8201		0.8413	0.038*	
C40	0.93562 (19)		0.81318 (16)	0.0319 (5)	
H40	0.9777		0.8710	0.038*	
C41	0.96730 (17)		0.74663 (16)	0.0287 (5)	
H41	1.0310		0.7590	0.034*	
C42	0.90628 (16)		0.66178 (15)	0.0225 (4)	
H42	0.9281		0.6168	0.027*	
O1	0.40792 (18)		0.82446 (14)	0.0565 (5)	
C43	0.44804 (19)		0.90584 (17)	0.0368 (6)	
C44	0.50130 (18)		0.92599 (16)	0.0339 (5)	
C45	0.55287 (18)		1.01771 (17)	0.0345 (5)	
C46	0.6016 (2)		1.03389 (19)	0.0461 (7)	
H46	0.6360		1.0951	0.055*	
C47	0.6028 (2)		0.9611 (2)	0.0513 (7)	
H47	0.6372		0.9730	0.062*	
C48	0.5518 (2)		0.8727 (2)	0.0460 (6)	
H48	0.5495		0.8238	0.055*	
C49	0.50413 (19)		0.85410 (16)	0.0323 (5)	
H49	0.4733		0.7918	0.039*	

### cyclo-Dodecakis(µ2-phenylmethanethiolato-κ2S:S)hexapalladium(6 Pd—Pd)–anthracene-9,10-dione (1/1) (mo_b0283_0m) Atomic displacement parameters (Å^2^)

**Table:**

	*U*^11^	*U*^22^	*U*^33^	*U*^12^	*U*^13^	*U*^23^
Pd1	0.01360 (7)	0.01177 (6)	0.01328 (6)	0.00124 (5)	0.00019 (5)	0.00521 (5)
Pd2	0.01367 (7)	0.01141 (6)	0.01350 (6)	0.00108 (5)	−0.00006 (5)	0.00477 (5)
Pd3	0.01424 (7)	0.01067 (6)	0.01404 (7)	0.00100 (5)	0.00019 (5)	0.00438 (5)
S1	0.0155 (2)	0.0137 (2)	0.0150 (2)	0.00133 (16)	0.00174 (16)	0.00482 (16)
S2	0.0157 (2)	0.0155 (2)	0.0145 (2)	0.00187 (16)	0.00014 (16)	0.00552 (16)
S3	0.0163 (2)	0.0148 (2)	0.0164 (2)	0.00159 (16)	0.00165 (16)	0.00749 (16)
S4	0.0163 (2)	0.0140 (2)	0.0142 (2)	0.00107 (16)	−0.00068 (16)	0.00557 (16)
S5	0.0169 (2)	0.01204 (19)	0.0169 (2)	0.00067 (16)	0.00173 (16)	0.00513 (16)
S6	0.0175 (2)	0.0136 (2)	0.0157 (2)	0.00024 (16)	−0.00150 (16)	0.00665 (16)
C1	0.0190 (9)	0.0196 (9)	0.0184 (9)	0.0021 (7)	0.0018 (7)	0.0092 (7)
C2	0.0168 (9)	0.0238 (9)	0.0170 (9)	0.0006 (7)	−0.0003 (7)	0.0106 (7)
C3	0.0221 (10)	0.0253 (10)	0.0210 (10)	0.0006 (8)	−0.0003 (8)	0.0064 (8)
C4	0.0321 (12)	0.0398 (13)	0.0195 (10)	0.0128 (10)	0.0042 (8)	0.0085 (9)
C5	0.0225 (11)	0.0615 (16)	0.0314 (12)	0.0134 (10)	0.0113 (9)	0.0301 (12)
C6	0.0178 (10)	0.0461 (14)	0.0401 (13)	−0.0043 (9)	−0.0012 (9)	0.0306 (11)
C7	0.0221 (10)	0.0254 (10)	0.0246 (10)	−0.0031 (8)	−0.0029 (8)	0.0135 (8)
C8	0.0243 (10)	0.0144 (9)	0.0191 (9)	0.0036 (7)	−0.0010 (7)	0.0031 (7)
C9	0.0262 (10)	0.0123 (8)	0.0198 (9)	0.0027 (7)	−0.0018 (8)	0.0017 (7)
C10	0.0268 (11)	0.0276 (11)	0.0319 (11)	−0.0027 (9)	−0.0033 (9)	0.0089 (9)
C11	0.0315 (13)	0.0372 (13)	0.0557 (16)	−0.0077 (10)	−0.0188 (12)	0.0148 (12)
C12	0.0607 (18)	0.0309 (13)	0.0402 (14)	−0.0060 (12)	−0.0312 (13)	0.0081 (11)
C13	0.0710 (19)	0.0274 (12)	0.0195 (11)	0.0039 (12)	−0.0085 (11)	0.0020 (9)
C14	0.0393 (12)	0.0232 (10)	0.0203 (10)	0.0076 (9)	0.0028 (9)	0.0029 (8)
C15	0.0225 (9)	0.0179 (9)	0.0170 (9)	0.0036 (7)	0.0036 (7)	0.0076 (7)
C16	0.0202 (9)	0.0180 (9)	0.0146 (8)	0.0011 (7)	−0.0029 (7)	0.0067 (7)
C17	0.0230 (10)	0.0251 (10)	0.0180 (9)	0.0002 (8)	−0.0010 (7)	0.0090 (8)
C18	0.0390 (12)	0.0334 (12)	0.0173 (9)	−0.0142 (10)	−0.0016 (9)	0.0104 (9)
C19	0.0666 (17)	0.0205 (10)	0.0180 (10)	−0.0128 (10)	−0.0086 (10)	0.0099 (8)
C20	0.0575 (15)	0.0172 (10)	0.0192 (10)	0.0096 (10)	−0.0094 (10)	0.0051 (8)
C21	0.0314 (11)	0.0207 (10)	0.0183 (9)	0.0063 (8)	−0.0022 (8)	0.0062 (8)
C22	0.0169 (9)	0.0187 (9)	0.0179 (9)	0.0002 (7)	0.0000 (7)	0.0082 (7)
C23	0.0165 (9)	0.0203 (9)	0.0156 (8)	−0.0013 (7)	0.0032 (7)	0.0077 (7)
C24	0.0254 (10)	0.0256 (10)	0.0224 (10)	0.0019 (8)	0.0007 (8)	0.0108 (8)
C25	0.0325 (12)	0.0444 (14)	0.0322 (12)	−0.0009 (10)	−0.0055 (9)	0.0243 (11)
C26	0.0393 (13)	0.0556 (16)	0.0218 (11)	−0.0131 (12)	−0.0089 (9)	0.0186 (11)
C27	0.0419 (13)	0.0369 (13)	0.0187 (10)	−0.0124 (10)	0.0015 (9)	0.0000 (9)
C28	0.0284 (11)	0.0220 (10)	0.0223 (10)	−0.0013 (8)	0.0041 (8)	0.0042 (8)
C29	0.0146 (9)	0.0156 (9)	0.0286 (10)	0.0010 (7)	0.0001 (7)	0.0089 (8)
C30	0.0127 (8)	0.0150 (8)	0.0221 (9)	0.0014 (7)	0.0036 (7)	0.0079 (7)
C31	0.0222 (10)	0.0226 (10)	0.0234 (10)	−0.0014 (8)	−0.0026 (8)	0.0113 (8)
C32	0.0318 (11)	0.0228 (10)	0.0233 (10)	−0.0062 (8)	−0.0042 (8)	0.0042 (8)
C33	0.0271 (10)	0.0141 (9)	0.0324 (11)	−0.0021 (8)	0.0020 (8)	0.0066 (8)
C34	0.0259 (10)	0.0201 (10)	0.0294 (10)	−0.0001 (8)	−0.0004 (8)	0.0141 (8)
C35	0.0231 (10)	0.0181 (9)	0.0212 (9)	−0.0033 (7)	−0.0032 (7)	0.0081 (8)
C36	0.0252 (10)	0.0139 (9)	0.0214 (9)	0.0035 (7)	−0.0040 (8)	0.0051 (7)
C37	0.0203 (9)	0.0158 (9)	0.0197 (9)	0.0054 (7)	−0.0010 (7)	0.0062 (7)
C38	0.0268 (11)	0.0240 (10)	0.0296 (11)	0.0023 (8)	−0.0008 (8)	0.0136 (9)
C39	0.0426 (13)	0.0329 (12)	0.0248 (11)	0.0110 (10)	0.0012 (9)	0.0161 (9)
C40	0.0358 (12)	0.0333 (12)	0.0226 (10)	0.0122 (10)	−0.0092 (9)	0.0046 (9)
C41	0.0213 (10)	0.0270 (11)	0.0325 (11)	0.0030 (8)	−0.0057 (8)	0.0036 (9)
C42	0.0207 (10)	0.0217 (10)	0.0250 (10)	0.0042 (8)	0.0008 (8)	0.0075 (8)
O1	0.0614 (13)	0.0745 (15)	0.0344 (10)	−0.0014 (11)	−0.0080 (9)	0.0205 (10)
C43	0.0286 (12)	0.0586 (16)	0.0255 (11)	−0.0101 (11)	−0.0076 (9)	0.0186 (11)
C44	0.0256 (11)	0.0528 (15)	0.0261 (11)	−0.0107 (10)	−0.0017 (9)	0.0181 (10)
C45	0.0242 (11)	0.0483 (14)	0.0310 (12)	−0.0096 (10)	0.0036 (9)	0.0140 (11)
C46	0.0350 (13)	0.080 (2)	0.0313 (13)	−0.0133 (13)	−0.0103 (10)	0.0308 (14)
C47	0.0313 (14)	0.088 (2)	0.0444 (16)	0.0005 (14)	0.0021 (11)	0.0351 (16)
C48	0.0423 (15)	0.0525 (16)	0.0411 (14)	−0.0083 (12)	0.0014 (12)	0.0136 (13)
C49	0.0310 (12)	0.0455 (14)	0.0190 (10)	−0.0032 (10)	−0.0067 (9)	0.0099 (9)

### cyclo-Dodecakis(µ2-phenylmethanethiolato-κ2S:S)hexapalladium(6 Pd—Pd)–anthracene-9,10-dione (1/1) (mo_b0283_0m) Geometric parameters (Å, º)

**Table:**

Pd1—Pd2	3.1609 (2)	C19—H19	0.9500
Pd1—Pd3^i^	3.1139 (2)	C19—C20	1.381 (4)
Pd1—S1	2.3231 (5)	C20—H20	0.9500
Pd1—S2	2.3374 (5)	C20—C21	1.384 (3)
Pd1—S3	2.3281 (5)	C21—H21	0.9500
Pd1—S6^i^	2.3264 (5)	C22—H22A	0.9900
Pd2—Pd3	3.0892 (2)	C22—H22B	0.9900
Pd2—S2	2.3342 (5)	C22—C23	1.504 (2)
Pd2—S3	2.3154 (4)	C23—C24	1.394 (3)
Pd2—S4	2.3250 (4)	C23—C28	1.387 (3)
Pd2—S5	2.3277 (5)	C24—H24	0.9500
Pd3—Pd1^i^	3.1139 (2)	C24—C25	1.387 (3)
Pd3—S1^i^	2.3367 (5)	C25—H25	0.9500
Pd3—S4	2.3230 (5)	C25—C26	1.382 (4)
Pd3—S5	2.3197 (4)	C26—H26	0.9500
Pd3—S6	2.3264 (5)	C26—C27	1.380 (4)
S1—Pd3^i^	2.3367 (5)	C27—H27	0.9500
S1—C1	1.8402 (18)	C27—C28	1.390 (3)
S2—C8	1.8440 (19)	C28—H28	0.9500
S3—C15	1.8378 (19)	C29—H29A	0.9900
S4—C22	1.8411 (19)	C29—H29B	0.9900
S5—C29	1.8365 (19)	C29—C30	1.507 (2)
S6—Pd1^i^	2.3264 (5)	C30—C31	1.385 (3)
S6—C36	1.8434 (19)	C30—C35	1.396 (3)
C1—H1A	0.9900	C31—H31	0.9500
C1—H1B	0.9900	C31—C32	1.391 (3)
C1—C2	1.501 (3)	C32—H32	0.9500
C2—C3	1.398 (3)	C32—C33	1.383 (3)
C2—C7	1.392 (3)	C33—H33	0.9500
C3—H3	0.9500	C33—C34	1.384 (3)
C3—C4	1.386 (3)	C34—H34	0.9500
C4—H4	0.9500	C34—C35	1.385 (3)
C4—C5	1.390 (4)	C35—H35	0.9500
C5—H5	0.9500	C36—H36A	0.9900
C5—C6	1.376 (4)	C36—H36B	0.9900
C6—H6	0.9500	C36—C37	1.505 (3)
C6—C7	1.392 (3)	C37—C38	1.393 (3)
C7—H7	0.9500	C37—C42	1.389 (3)
C8—H8A	0.9900	C38—H38	0.9500
C8—H8B	0.9900	C38—C39	1.394 (3)
C8—C9	1.501 (3)	C39—H39	0.9500
C9—C10	1.394 (3)	C39—C40	1.382 (4)
C9—C14	1.396 (3)	C40—H40	0.9500
C10—H10	0.9500	C40—C41	1.383 (3)
C10—C11	1.384 (3)	C41—H41	0.9500
C11—H11	0.9500	C41—C42	1.389 (3)
C11—C12	1.384 (4)	C42—H42	0.9500
C12—H12	0.9500	O1—C43	1.240 (3)
C12—C13	1.382 (4)	C43—C44	1.470 (4)
C13—H13	0.9500	C43—C45^ii^	1.473 (4)
C13—C14	1.384 (3)	C44—C45	1.425 (3)
C14—H14	0.9500	C44—C49	1.383 (3)
C15—H15A	0.9900	C45—C43^ii^	1.473 (4)
C15—H15B	0.9900	C45—C46	1.354 (4)
C15—C16	1.501 (2)	C46—H46	0.9500
C16—C17	1.393 (3)	C46—C47	1.436 (5)
C16—C21	1.394 (3)	C47—H47	0.9500
C17—H17	0.9500	C47—C48	1.387 (4)
C17—C18	1.391 (3)	C48—H48	0.9500
C18—H18	0.9500	C48—C49	1.389 (4)
C18—C19	1.383 (4)	C49—H49	0.9500
			
Pd3^i^—Pd1—Pd2	122.696 (6)	C16—C15—S3	109.37 (13)
S1—Pd1—Pd2	133.948 (12)	C16—C15—H15A	109.8
S1—Pd1—Pd3^i^	48.255 (11)	C16—C15—H15B	109.8
S1—Pd1—S2	99.152 (16)	C17—C16—C15	120.28 (17)
S1—Pd1—S3	178.778 (16)	C17—C16—C21	118.90 (18)
S1—Pd1—S6^i^	81.998 (16)	C21—C16—C15	120.82 (18)
S2—Pd1—Pd2	47.378 (11)	C16—C17—H17	119.8
S2—Pd1—Pd3^i^	129.132 (12)	C18—C17—C16	120.5 (2)
S3—Pd1—Pd2	46.933 (11)	C18—C17—H17	119.8
S3—Pd1—Pd3^i^	132.454 (13)	C17—C18—H18	119.9
S3—Pd1—S2	81.033 (16)	C19—C18—C17	120.1 (2)
S6^i^—Pd1—Pd2	128.215 (13)	C19—C18—H18	119.9
S6^i^—Pd1—Pd3^i^	47.991 (11)	C18—C19—H19	120.2
S6^i^—Pd1—S2	174.139 (16)	C20—C19—C18	119.59 (19)
S6^i^—Pd1—S3	97.940 (16)	C20—C19—H19	120.2
Pd3—Pd2—Pd1	121.425 (6)	C19—C20—H20	119.6
S2—Pd2—Pd1	47.463 (11)	C19—C20—C21	120.8 (2)
S2—Pd2—Pd3	128.222 (12)	C21—C20—H20	119.6
S3—Pd2—Pd1	47.270 (12)	C16—C21—H21	119.9
S3—Pd2—Pd3	131.984 (12)	C20—C21—C16	120.1 (2)
S3—Pd2—S2	81.367 (16)	C20—C21—H21	119.9
S3—Pd2—S4	177.813 (17)	S4—C22—H22A	110.0
S3—Pd2—S5	98.089 (16)	S4—C22—H22B	110.0
S4—Pd2—Pd1	134.849 (12)	H22A—C22—H22B	108.4
S4—Pd2—Pd3	48.318 (11)	C23—C22—S4	108.39 (12)
S4—Pd2—S2	99.980 (16)	C23—C22—H22A	110.0
S4—Pd2—S5	80.756 (16)	C23—C22—H22B	110.0
S5—Pd2—Pd1	128.271 (13)	C24—C23—C22	120.23 (17)
S5—Pd2—Pd3	48.229 (11)	C28—C23—C22	120.67 (17)
S5—Pd2—S2	173.880 (17)	C28—C23—C24	119.10 (18)
Pd2—Pd3—Pd1^i^	115.879 (6)	C23—C24—H24	119.8
S1^i^—Pd3—Pd1^i^	47.884 (11)	C25—C24—C23	120.4 (2)
S1^i^—Pd3—Pd2	130.174 (12)	C25—C24—H24	119.8
S4—Pd3—Pd1^i^	132.157 (12)	C24—C25—H25	120.0
S4—Pd3—Pd2	48.373 (11)	C26—C25—C24	119.9 (2)
S4—Pd3—S1^i^	178.547 (16)	C26—C25—H25	120.0
S4—Pd3—S6	99.246 (16)	C25—C26—H26	119.9
S5—Pd3—Pd1^i^	125.043 (13)	C27—C26—C25	120.1 (2)
S5—Pd3—Pd2	48.449 (12)	C27—C26—H26	119.9
S5—Pd3—S1^i^	97.903 (16)	C26—C27—H27	120.0
S5—Pd3—S4	80.964 (16)	C26—C27—C28	120.1 (2)
S5—Pd3—S6	169.831 (17)	C28—C27—H27	120.0
S6—Pd3—Pd1^i^	47.990 (11)	C23—C28—C27	120.3 (2)
S6—Pd3—Pd2	124.764 (13)	C23—C28—H28	119.8
S6—Pd3—S1^i^	81.707 (16)	C27—C28—H28	119.8
Pd1—S1—Pd3^i^	83.862 (15)	S5—C29—H29A	109.2
C1—S1—Pd1	109.07 (6)	S5—C29—H29B	109.2
C1—S1—Pd3^i^	111.18 (6)	H29A—C29—H29B	107.9
Pd2—S2—Pd1	85.159 (15)	C30—C29—S5	111.89 (13)
C8—S2—Pd1	103.40 (6)	C30—C29—H29A	109.2
C8—S2—Pd2	106.26 (7)	C30—C29—H29B	109.2
Pd2—S3—Pd1	85.797 (15)	C31—C30—C29	119.56 (17)
C15—S3—Pd1	110.88 (7)	C31—C30—C35	118.78 (17)
C15—S3—Pd2	111.96 (6)	C35—C30—C29	121.64 (17)
Pd3—S4—Pd2	83.308 (15)	C30—C31—H31	119.8
C22—S4—Pd2	111.99 (6)	C30—C31—C32	120.31 (18)
C22—S4—Pd3	112.72 (6)	C32—C31—H31	119.8
Pd3—S5—Pd2	83.321 (15)	C31—C32—H32	119.7
C29—S5—Pd2	103.36 (6)	C33—C32—C31	120.59 (19)
C29—S5—Pd3	109.93 (6)	C33—C32—H32	119.7
Pd1^i^—S6—Pd3	84.020 (15)	C32—C33—H33	120.3
C36—S6—Pd1^i^	108.12 (6)	C32—C33—C34	119.45 (18)
C36—S6—Pd3	106.45 (7)	C34—C33—H33	120.3
S1—C1—H1A	109.6	C33—C34—H34	119.9
S1—C1—H1B	109.6	C33—C34—C35	120.10 (18)
H1A—C1—H1B	108.1	C35—C34—H34	119.9
C2—C1—S1	110.14 (13)	C30—C35—H35	119.6
C2—C1—H1A	109.6	C34—C35—C30	120.75 (18)
C2—C1—H1B	109.6	C34—C35—H35	119.6
C3—C2—C1	120.30 (17)	S6—C36—H36A	109.9
C7—C2—C1	121.12 (18)	S6—C36—H36B	109.9
C7—C2—C3	118.57 (18)	H36A—C36—H36B	108.3
C2—C3—H3	119.7	C37—C36—S6	108.93 (13)
C4—C3—C2	120.7 (2)	C37—C36—H36A	109.9
C4—C3—H3	119.7	C37—C36—H36B	109.9
C3—C4—H4	120.0	C38—C37—C36	120.57 (18)
C3—C4—C5	120.0 (2)	C42—C37—C36	120.61 (17)
C5—C4—H4	120.0	C42—C37—C38	118.81 (18)
C4—C5—H5	120.1	C37—C38—H38	119.8
C6—C5—C4	119.9 (2)	C37—C38—C39	120.4 (2)
C6—C5—H5	120.1	C39—C38—H38	119.8
C5—C6—H6	119.9	C38—C39—H39	119.9
C5—C6—C7	120.3 (2)	C40—C39—C38	120.1 (2)
C7—C6—H6	119.9	C40—C39—H39	119.9
C2—C7—C6	120.6 (2)	C39—C40—H40	120.1
C2—C7—H7	119.7	C39—C40—C41	119.8 (2)
C6—C7—H7	119.7	C41—C40—H40	120.1
S2—C8—H8A	109.5	C40—C41—H41	119.9
S2—C8—H8B	109.5	C40—C41—C42	120.2 (2)
H8A—C8—H8B	108.0	C42—C41—H41	119.9
C9—C8—S2	110.92 (13)	C37—C42—H42	119.7
C9—C8—H8A	109.5	C41—C42—C37	120.6 (2)
C9—C8—H8B	109.5	C41—C42—H42	119.7
C10—C9—C8	120.45 (18)	O1—C43—C44	120.5 (3)
C10—C9—C14	119.15 (19)	O1—C43—C45^ii^	120.3 (3)
C14—C9—C8	120.40 (19)	C44—C43—C45^ii^	119.2 (2)
C9—C10—H10	120.0	C45—C44—C43	120.8 (2)
C11—C10—C9	120.1 (2)	C49—C44—C43	119.5 (2)
C11—C10—H10	120.0	C49—C44—C45	119.6 (2)
C10—C11—H11	119.8	C44—C45—C43^ii^	120.0 (2)
C10—C11—C12	120.5 (3)	C46—C45—C43^ii^	120.6 (2)
C12—C11—H11	119.8	C46—C45—C44	119.4 (2)
C11—C12—H12	120.1	C45—C46—H46	119.1
C13—C12—C11	119.8 (2)	C45—C46—C47	121.9 (2)
C13—C12—H12	120.1	C47—C46—H46	119.1
C12—C13—H13	119.9	C46—C47—H47	121.4
C12—C13—C14	120.2 (2)	C48—C47—C46	117.2 (3)
C14—C13—H13	119.9	C48—C47—H47	121.4
C9—C14—H14	119.9	C47—C48—H48	119.2
C13—C14—C9	120.3 (2)	C47—C48—C49	121.6 (3)
C13—C14—H14	119.9	C49—C48—H48	119.2
S3—C15—H15A	109.8	C44—C49—C48	120.2 (2)
S3—C15—H15B	109.8	C44—C49—H49	119.9
H15A—C15—H15B	108.2	C48—C49—H49	119.9
			
Pd1—S1—C1—C2	152.50 (12)	C17—C18—C19—C20	−1.1 (3)
Pd1—S2—C8—C9	−170.66 (13)	C18—C19—C20—C21	1.3 (3)
Pd1—S3—C15—C16	121.75 (12)	C19—C20—C21—C16	−0.2 (3)
Pd1^i^—S6—C36—C37	175.62 (12)	C21—C16—C17—C18	1.1 (3)
Pd2—S2—C8—C9	−81.85 (14)	C22—C23—C24—C25	−179.93 (19)
Pd2—S3—C15—C16	−144.29 (11)	C22—C23—C28—C27	179.26 (19)
Pd2—S4—C22—C23	−132.34 (11)	C23—C24—C25—C26	0.8 (3)
Pd2—S5—C29—C30	−60.03 (14)	C24—C23—C28—C27	−1.1 (3)
Pd3^i^—S1—C1—C2	−116.78 (12)	C24—C25—C26—C27	−1.4 (4)
Pd3—S4—C22—C23	135.78 (11)	C25—C26—C27—C28	0.7 (4)
Pd3—S5—C29—C30	−147.68 (12)	C26—C27—C28—C23	0.5 (3)
Pd3—S6—C36—C37	86.64 (13)	C28—C23—C24—C25	0.5 (3)
S1—C1—C2—C3	−78.1 (2)	C29—C30—C31—C32	177.18 (19)
S1—C1—C2—C7	102.25 (18)	C29—C30—C35—C34	−177.65 (18)
S2—C8—C9—C10	93.6 (2)	C30—C31—C32—C33	0.6 (3)
S2—C8—C9—C14	−85.6 (2)	C31—C30—C35—C34	0.9 (3)
S3—C15—C16—C17	125.44 (16)	C31—C32—C33—C34	0.7 (3)
S3—C15—C16—C21	−54.9 (2)	C32—C33—C34—C35	−1.2 (3)
S4—C22—C23—C24	−89.47 (19)	C33—C34—C35—C30	0.4 (3)
S4—C22—C23—C28	90.15 (19)	C35—C30—C31—C32	−1.4 (3)
S5—C29—C30—C31	139.77 (16)	C36—C37—C38—C39	−179.55 (19)
S5—C29—C30—C35	−41.7 (2)	C36—C37—C42—C41	−179.97 (18)
S6—C36—C37—C38	92.23 (19)	C37—C38—C39—C40	−0.2 (3)
S6—C36—C37—C42	−86.4 (2)	C38—C37—C42—C41	1.3 (3)
C1—C2—C3—C4	−179.22 (18)	C38—C39—C40—C41	0.9 (3)
C1—C2—C7—C6	179.76 (18)	C39—C40—C41—C42	−0.4 (3)
C2—C3—C4—C5	−0.8 (3)	C40—C41—C42—C37	−0.7 (3)
C3—C2—C7—C6	0.1 (3)	C42—C37—C38—C39	−0.9 (3)
C3—C4—C5—C6	0.6 (3)	O1—C43—C44—C45	176.5 (2)
C4—C5—C6—C7	−0.1 (3)	O1—C43—C44—C49	−1.6 (4)
C5—C6—C7—C2	−0.3 (3)	C43—C44—C45—C43^ii^	0.8 (4)
C7—C2—C3—C4	0.5 (3)	C43—C44—C45—C46	−179.7 (2)
C8—C9—C10—C11	179.8 (2)	C43—C44—C49—C48	−178.4 (2)
C8—C9—C14—C13	−179.77 (19)	C43^ii^—C45—C46—C47	179.3 (2)
C9—C10—C11—C12	0.5 (4)	C44—C45—C46—C47	−0.1 (4)
C10—C9—C14—C13	1.0 (3)	C45^ii^—C43—C44—C45	−0.8 (4)
C10—C11—C12—C13	0.0 (4)	C45^ii^—C43—C44—C49	−178.9 (2)
C11—C12—C13—C14	0.0 (4)	C45—C44—C49—C48	3.5 (4)
C12—C13—C14—C9	−0.6 (3)	C45—C46—C47—C48	0.0 (4)
C14—C9—C10—C11	−1.0 (3)	C46—C47—C48—C49	1.8 (4)
C15—C16—C17—C18	−179.22 (18)	C47—C48—C49—C44	−3.6 (4)
C15—C16—C21—C20	179.37 (18)	C49—C44—C45—C43^ii^	178.9 (2)
C16—C17—C18—C19	−0.1 (3)	C49—C44—C45—C46	−1.7 (3)
C17—C16—C21—C20	−0.9 (3)		

Symmetry codes: (i) −*x*+1, −*y*+1, −*z*+1; (ii) −*x*+1, −*y*+1, −*z*+2.
